# Can data from native mosquitoes support determining invasive species habitats? Modelling the climatic niche of *Aedes japonicus japonicus* (Diptera, Culicidae) in Germany

**DOI:** 10.1007/s00436-019-06513-5

**Published:** 2019-11-26

**Authors:** Antje Kerkow, Ralf Wieland, Linus Früh, Franz Hölker, Jonathan M. Jeschke, Doreen Werner, Helge Kampen

**Affiliations:** 1 Leibniz Centre for Agricultural Landscape Research (ZALF), Eberswalder Str. 84, 15374 Müncheberg, Germany; 2 Department of Biology, Chemistry, Pharmacy, Institute of Biology, Freie Universität Berlin, Königin-Luise-Str. 1-3, 14195 Berlin, Germany; 3 Leibniz-Institute of Freshwater Ecology and Inland Fisheries (IGB), Müggelseedamm 310, 12587 Berlin, Germany; 4 Berlin-Brandenburg Institute of Advanced Biodiversity Research (BBIB), Altensteinstr. 34, 14195 Berlin, Germany; 5 Friedrich-Loeffler-Institut (FLI), Federal Research Institute for Animal Health, Südufer 10, 17493 Greifswald - Insel Riems, Germany

**Keywords:** Citizen science, Invasive species distribution models, Machine learning, Occurrence probability, Support vector machine

## Abstract

Invasive mosquito species and the pathogens they transmit represent a serious health risk to both humans and animals. Thus, predictions on their potential geographic distribution are urgently needed. In the case of a recently invaded region, only a small number of occurrence data is typically available for analysis, and absence data are not reliable. To overcome this problem, we have tested whether it is possible to determine the climatic ecological niche of an invasive mosquito species by using both the occurrence data of other, native species and machine learning. The approach is based on a support vector machine and in this scenario applied to the Asian bush mosquito (*Aedes japonicus japonicus*) in Germany. Presence data for this species (recorded in the Germany since 2008) as well as for three native mosquito species were used to model the potential distribution of the invasive species. We trained the model with data collected from 2011 to 2014 and compared our predicted occurrence probabilities for 2015 with observations found in the field throughout 2015 to evaluate our approach. The prediction map showed a high degree of concordance with the field data. We applied the model to medium climate conditions at an early stage of the invasion (2011–2015), and developed an explanation for declining population densities in an area in northern Germany. In addition to the already known distribution areas, our model also indicates a possible spread to Saarland, southwestern Rhineland-Palatinate and in 2015 to southern Bavaria, where the species is now being increasingly detected. However, there is also evidence that the possible distribution area under the mean climate conditions was underestimated.

## Introduction

Due to globalisation, facilitating long-distance traffic, mass tourism and worldwide trade, increasing numbers of invasive mosquitoes have recently arrived and subsequently established themselves in Germany and mainland Europe (Medlock et al. 2015). As they include potential vectors of a wide range of human and animal pathogens (Schaffner et al. 2013; Becker et al. 2014), they have become a major research issue. To estimate the risk and take protective measures against mosquito-borne disease outbreaks, it is of utmost importance to know the suitable habitats of the various vector species.

We think that the habitats of invasive species can be analysed most effectively in a stepwise procedure. The first step should be to identify the climatic niche within the invaded area. This is challenging because the spread of invasive species (especially short-lived exothermic insects) is influenced by spontaneous weather events on the one hand, but also by long-term climate, which has impacted vegetation and mosquito populations in the past. In the second step, landscape elements such as land use and altitude need to be considered. However, some regions, although suitable for an invasive species, are unlikely to become populated by active migration due to dispersal barriers. In the case of mosquitoes, for example, these can be large cultivated areas. On the other hand, there are propagation paths that enable rapid passive spread, for example, along roads and waterways, because some mosquito species may be displaced by cars in their adult forms (Eritja et al. 2017) or by container ships in their egg stages (Eritja et al. 2005; Hofhuis et al. 2008; Reiter 2010). Therefore, in the third step, the consideration of propagation paths is useful, including a propagation simulation.

In this paper, we focus on the first step and try to identify climatically suitable areas for the Asian bush mosquito (*Aedes japonicus japonicus* (Theobald, 1901)) in Germany.

The species is one of eight non-indigenous culicid species recently registered in Germany, and apparently the most widespread of them (Kampen et al. 2017; Koban et al. 2019). It originates from East Asia (Miyagi 1971) and was first recorded in 1998 and 2000 in North America and Europe. Since then, it has expanded its distribution range rapidly on both continents (Peyton et al. 1999; Kampen and Werner 2014; Kampen et al. 2017). In its native range as well as in most invasion areas, the climate is temperate and characterised by winters with frost and snowfall. However, it was also found in subtropical and tropical climates such as Florida and Hawaii (Egizi and Fonseca 2015; Riles et al. 2017). The eggs of this species are desiccation and frost resistant (Reuss et al. 2018) and are laid by the females in rock pools of rivers, water-filled tree holes or various kinds of small artificial containers that are able to collect water such as flower pots or vases, buckets, ash trays and bird baths (Tanaka et al. 1979; Scott 2003; Kampen et al. 2012; Kaufman et al. 2012). Egg hatching and larval development in spring begin at 4–5 °C, and development time decreases significantly with temperatures rising up to 28 °C (Scott 2003; Burger and Davis 2008; Kampen et al. 2016a). Temperatures above 34 °C inhibit larval development (Scott 2003). The species usually overwinters in the egg stage, but in warmer regions, it is also possible and observed to hibernate in the larval stage (Reuss et al. 2018; Bova et al. 2019). Immature stages are usually found both sooner in spring and later in autumn than coexisting mosquito species (Iriarte et al. 1991; Burger and Davis 2008; Kaufman and Fonseca 2014).

For habitat analysis of both invasive and native species, data-driven machine learning approaches have been widely applied and proven successful (Drake et al. 2006; Jeschke and Strayer 2008; Früh et al. 2018). In general, species distribution models can be created using either presence-only or combined presence-absence data. Absence data would doubtlessly improve the distribution predictions for an invasive species (Elith et al. 2006; Vaclavik and Meentemeyer 2009), and they can be obtained either via monitoring programmes or computer simulations. The simulated so-called background or pseudo-absence data are randomly distributed points in the entire model area or only in regions without evidence and with a certain spatial distance to the known occurrences (VanDerWal et al. 2009; Barbet-Massin et al. 2012).

When modelling invasive species that have only recently been established, the available dataset often poses a problem because usually only a small number of presence data are available. Furthermore, absence data cannot be used for modelling (Liu et al. 2013), as it is unknown whether the species is not present in a certain area because (i) the area does not offer appropriate habitat conditions, (ii) the area is not accessible due to dispersal barriers, or (iii) not enough time has passed for the species to arrive. Additionally (iv), it is possible that the species does occur in an area, even though it has not been found during field surveys. The latter may particularly be the case when very large areas are surveyed, as during the *Ae. j. japonicus* monitoring throughout Germany (Kampen et al. 2016a).

Although the use of background/pseudo-absence data leads to an artificially enforced high evaluation of a model and is therefore hotly debated (VanDerWal et al. 2009; Vaclavik and Meentemeyer 2009), the majority of invasive mosquito distribution models rely on them. Currently, the most frequently applied system of modelling is the maximum entropy (MaxEnt) modelling algorithm (Phillips et al. 2006; Fischer et al. 2011; Rochlin et al. 2013; Thomas et al. 2014; Melaun et al. 2015). Combinations of machine learning algorithms for presence and background data have also been used with the objective to obtain more robust predictions than with a single algorithm (Kraemer et al. 2015; Cunze et al. 2016). However, this procedure is also under discussion (Demertzis et al. 2017).

Predictive distribution maps based on either single or combined machine learning algorithms risk underestimating the future habitats of invasive species, as only a short time has passed since the arrival of the species. Furthermore, training data may not reflect all possible ranges under the selected climate variables due to lacking propagation paths between the suitable habitats. To circumvent these problems, scientists have used data from other countries and continents, where the mosquito species of interest is native or had already been present for a longer period of time (Fischer et al. 2011; Thomas et al. 2014). In this way, a large dataset can be provided. However, a given species may fill specific ecological niches in particular countries due to different environmental contexts (Jeschke and Strayer 2008). Therefore, using data of the native range may not always lead to accurate predictions for an invaded area.

Thanks to a nationwide monitoring programme established in Germany several years ago, which also includes the citizen science project “Mueckenatlas” (Werner et al. 2014; Kampen et al. 2015; Walther and Kampen 2017), a large amount of occurrence data is available concerning the German mosquito fauna, which includes around 50 species. Based on the idea that each species probably occupies its individual and unique ecological niche, we have tested whether it is possible to distinguish the climatic niche of *Ae. j. japonicus* from the climatic niches of native species. Thus, contrary to modelling approaches relying on occurrence data outside the model region, as well as to a modelling approach based on life history data obtained from laboratory analyses (Wieser et al. 2019), we aim to determine the realised ecological niche (Jeschke and Strayer 2008). This was achieved by the application of a machine learning approach based on a support vector machine (Cortes and Vapnik 1995), presence data of *Ae. j. japonicu*s and three native mosquito species from the invaded region (Germany). The algorithm calculates a dividing hyperplane for the vector dataset of environmental parameters and for the four mosquito species, respectively. It is not easy to identify which weather data explain the distribution patterns of the species at best. We have therefore developed a procedure in the framework of this study, which has been published separately (Wieland et al. 2017).

The support vector machine (Cortes and Vapnik 1995) is a supervised learning algorithm and frequently used for data-driven species distribution models (e.g. Drake et al. 2006; Fukuda et al. 2013). It was developed in the 1990s along with a number of other machine learning techniques such as regression tree and random forest. The algorithm was primarily implemented as a linear binary classifier that maximises the margin between two sampling groups (Cortes and Vapnik 1995; Kampichler et al. 2010). When calculating the exact position and orientation of the hyperplane that separates the groups from each other, the algorithm does not consider all data points, but only those closest to the plane, the so-called supported vectors. Advanced forms of support vector machines use non-linear kernels (Nalepa and Kawulok 2019). By means of the “kernel-trick”, the training data are mapped into a higher dimensional space which simplifies the computation of the separating hyperplanes for complicated patterns (Drake et al. 2006; Kampichler et al. 2010; Nalepa and Kawulok 2019). A kernel of the RBF (radial basis function) type was used in this approach.

Research by our working group (Früh et al. 2018) has shown that even with noisy data, the application of the support vector machine in combination with a low-dimensional space-kernel is relatively robust against over-fitting. The algorithm is thus a suitable method of machine learning for our relatively small data sets which are prone to this issue. Früh et al. (2018) found that the algorithm did not always achieve the highest accuracy in training, but often came out best in validation. The support vector machine as a classifier produces binary outputs, but probability values can be generated in a post-processing step (Platt 1999). These probabilities give a much more detailed picture of the spatial extent of the habitat than binary values.

In summary, the aims of this study are (i) to develop a model dependant on climate parameters that enables accurate predictions of future distributions of an invasive mosquito species based on a limited amount of presence-only data, and (ii) to apply this model to *Ae. j. japonicus* in Germany.

## Materials and methods

### Native species

For habitat separation, we have selected two native mosquito species that are widespread and common throughout Germany: *Aedes vexans* (Meigen, 1830) and *Aedes geniculatus* (Olivier, 1791)*. Aedes vexans* is a floodwater species of the lowlands, where it develops in temporary water bodies. It is adapted to temperate climates (Becker et al. 2010) and develops in tremendous numbers after heavy rainfall and flooding. Larval hatching starts at temperatures above 9 °C, but development is optimal at 30 °C. As with *Ae. j. japonicus*, the eggs are resistant against drought and frost and can even sustain temperatures as low as − 20 °C (Becker et al. 2010).

*Aedes geniculatus* preferably breeds in water-filled tree holes, open tree stumps and branch axils that can be found on mature, deciduous trees (Dahl and Blackmore 2001). Eggs are laid on humid walls of the wood, and the larvae hatch after rainwater has filled the holes (Dahl and Blackmore 2001). Like the Asian bush mosquito, *Aedes geniculatus* overwinters either in the egg stage in cold regions or in the larval stage in warmer regions and the eggs can withstand freezing.

We additionally picked one species, *Anopheles daciae* Linton, Nicolescu and Harbach 2004, whose occurrence is particularly concentrated in the north-eastern part of Germany in order to take account of particular continental conditions in Germany. *Anopheles daciae* is a member of the *Anopheles maculipennis* complex, which has only recently been described (Nicolescu et al. 2004). Little is known yet about the ecology of this species. In the framework of a nationwide mosquito monitoring programme in Germany, *An. daciae* was found in the northern Upper Rhine Valley, in the eastern North German lowlands and in the lower river valleys in southern Germany (Kampen et al. 2016b). This indicates linkage of occurrence to both low altitude and relatively continental climate conditions.

**Fig. 1:**
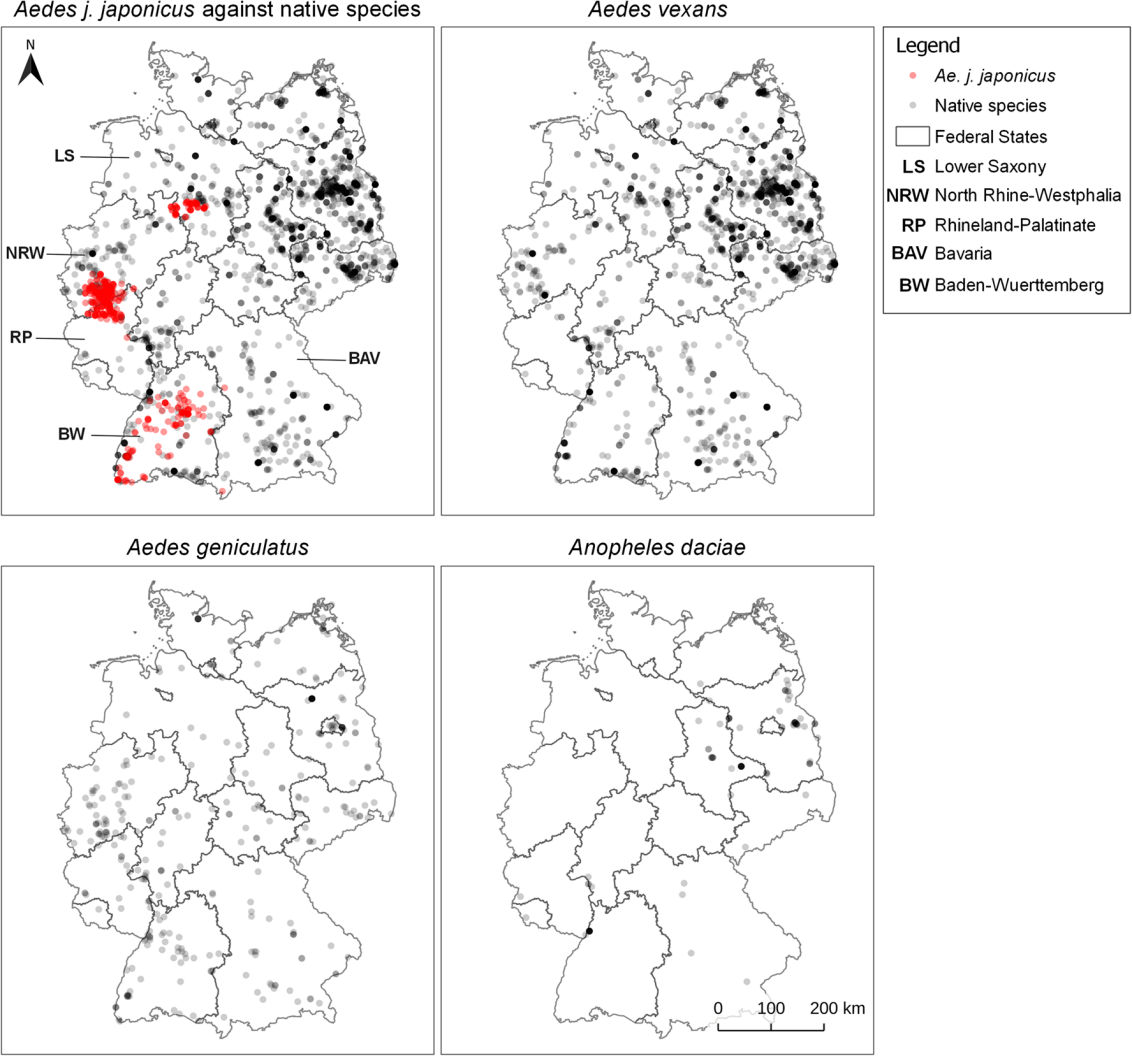
Visualisation of species sampling data of the years 2011–2014 (training years for the model). The evidence points appear in high colour intensity when several of the same colour overlay each other. This image was created with QGIS 3.8.2

Figure 1 shows the collection sites of each model species in Germany from 2011 to 2014, based on nationwide systematic active and passive mosquito monitoring. The passive monitoring data originate from the “Mueckenatlas” project and account for 68.9% of the data used for training and evaluation of this model. The active monitoring data are derived from on-site collections in regions from which invasive mosquito species were submitted, by examining possible breeding sites for larvae and installing mosquito traps.

### Data access

#### Mosquito database

The mosquito occurrence data are derived from monitoring projects carried out in Germany since 2011 and stored inside the German national mosquito database (CULBASE, https://culbase.fli.de/Public/Page/Info.aspx). Amongst others, the database contains collection sites (including coordinates), mode of collection (including dates) and methods of species determination. The CULBASE database can be accessed via an export interface that enables querying data of the species and the year of interest, and downloading these data as Excel files, one file for each species. Data of *Ae. j. japonicus*, *Ae. vexans*, *Ae. geniculatus* and *An. daciae* from the years 2011–2014 were filtered for training the model, and data from 2015 to evaluate it. The Excel files contained the coordinates and the dates of collection. These data were entered into our object-oriented model using the PYTHON “PANDAS” module.

#### Weather and climate data

We used freely available data from the German Weather Service (Deutscher Wetterdienst 2017) which are gridded for Germany with a cell size of 1 km × 1 km and provide hourly, daily, monthly, seasonal, yearly and multi-annual resolutions. In the following, we define data up to an annual resolution as weather data and multi-year averages as climate data.

For each of the four mosquito species and their occurrence points in the training years 2011, 2012, 2013 and 2014, we created Excel tables with the help of a PYTHON script. These tables were the input for training the model; they contained the weather data for each occurrence point. According to Wieland et al. (2017), we used the following eight weather variables: mean temperature in spring (March, April, May) [T13], September [T09], October [T10] and December [T12]; sum of precipitation in February [P02], April [P04] and June [P06]; and drought index of autumn (September, October, November) [D15]. Wieland et al. (2017) use the same model as presented here and describe a method for the analysis of weather data that explain the distribution of the mosquito species at best. Therefore, a set of 37 data was pre-selected that may play a role in a biological point of view. These were, for example, temperature and precipitation data during the breeding season and the temperature and number of frost days during the winter months. The data sets were reduced to the best combination using a genetic optimisation procedure. The optimisation parameter was the *f*1 score (see subsection “Model validation” for the definition).

### Model training

**Fig. 2:**
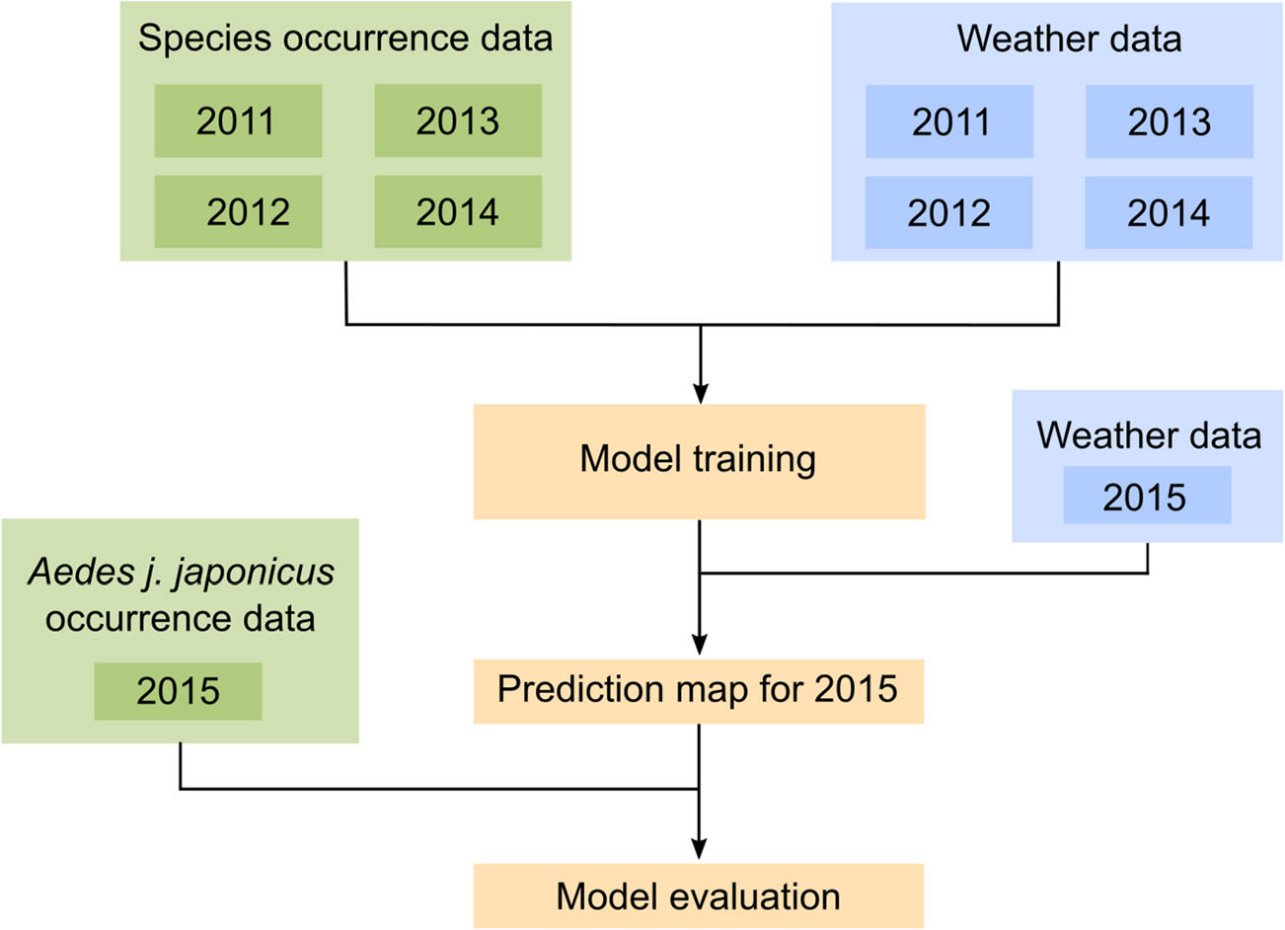
Workflow: Model training and validation. This image was created with Inkscape 0.92

As shown in Fig. 2, we used the species occurrence data and the weather data from 2011 to 2014, respectively, for training the model. The support vector machine only needed to distinguish between two classes, the invasive target species on the one hand, and the native species on the other hand. By combining the monitoring data of several native species to one class, we converted a multi-classification problem into a binary classification problem, which helps to correctly calculate a decision boundary for the target species in space (Garreta and Moncecchi 2013).

As a training algorithm, we used the support vector machine from the SCIKIT toolbox (Pedregosa et al. 2011). Our training dataset contained 508 occurrence points for *Ae. j. japonicus*, 2056 for *Ae. vexans*, 322 for *Ae. geniculatus* and 102 for *An. daciae*. These data and the stored weather conditions were read in from the Excel tables with the PYTHON “PANDAS” tool. To mitigate the unbalanced numbers of mosquito species data, a maximum of 1000 data points per species were randomly selected for training the model. We used a radial basis function (RBF) kernel and passed the following hyper-parameters as arguments to the constructor of the support vector machine: gamma = 0.0005, C = 1.0, tolerance = 1e-10, probability = true.

The support vector machine calculated the hyperplane for the separation of both classes. With the returned binary values for the training data (positive or negative), the confusion matrix was calculated. By re-training the parameters on a sigmoid function, the original outputs were mapped into probability values on a scale from 0 (very low probability of occurrence) to 1 (very high probability of occurrence) (Platt 1999; Pedregosa et al. 2011). This procedure is implemented by default in SCIKIT-LEARN. The latter training results were saved and used for creating distribution maps of *Ae. j. japonicus* under certain weather conditions.

#### Analysis of the training classes

To illustrate the different dependencies of the species on the eight weather variables, we presented the values for the two training classes (“*Ae. j. japonicus*” and “native species”) at their respective sampling sites in violin plots. We additionally tested the data of both groups for significant differences (*p* < 0.05) with the Wilcoxon-Mann-Whitney test, using R and the wilcox.test-function where we set the following parameters: paired = FALSE, alternative = “two.sided”.

### Model validation

#### Classification

The classification result was evaluated by calculating a confusion matrix and the values of precision, recall and *f*1 score. A confusion matrix summarises the reclassification results for the set of test data (Garreta and Moncecchi 2013). Based on this matrix, the values for precision and recall are calculated for the two classes (“*Ae. j. japonicus*” and “native species”). The precision computes the proportion of true positive values (TP) from the predicted positive values, including true positive and false positive (FP) values (Eq. 1). By contrast, the recall calculates what proportion of the positive observations was correctly evaluated. This calculation (Eq. 2) considers the false negative (FN) values.

1 $$ \mathrm{Precision}=\frac{\mathrm{TP}}{\mathrm{TP}+\mathrm{FP}} $$

2 $$ \mathrm{Recall}=\frac{\mathrm{TP}}{\mathrm{TP}+\mathrm{FN}} $$

The *f*1 score represents the harmonic mean of precision and recall (Eq. 3).

3 $$ f1=\frac{2\left(\mathrm{Prec}\cdotp \mathrm{Rec}\right)}{\mathrm{Prec}+\mathrm{Rec}} $$

To calculate the training results for both classes, weighted mean values were calculated for precision, recall and *f*1 score (Eq. 4). When *x* is the validation variable and *n* the sum of observations, then applies:

4 $$ \overline{x}=\frac{x_{\mathrm{japonicus}}\cdotp {n}_{\mathrm{japonicus}}+{x}_{\mathrm{native}}\cdotp {n}_{\mathrm{native}}}{n_{\mathrm{japonicus}}+{n}_{\mathrm{native}}}. $$

#### Predictive power

In order to validate the predictive power of our model, we used the *Ae. j. japonicus* sampling data of 2015, which had not been included in the training, and analysed the pixel values of the predictive distribution map, referring to the weather conditions of 2015. We drew a histogram and calculated the median and interquartile range to describe the pixel values for the validation dataset.

### Colonisation potential between 2011 and 2015

The weather conditions of individual years can differ significantly from one another and with regionally varying intensity, influencing the distribution potential of the mosquito species. We have therefore carried out a model application for the average climate values for the years 2011–2015 and produced a corresponding habitat map for *Ae. j japonicus*.

## Results

### Analysis of the training classes

**Fig. 3:**
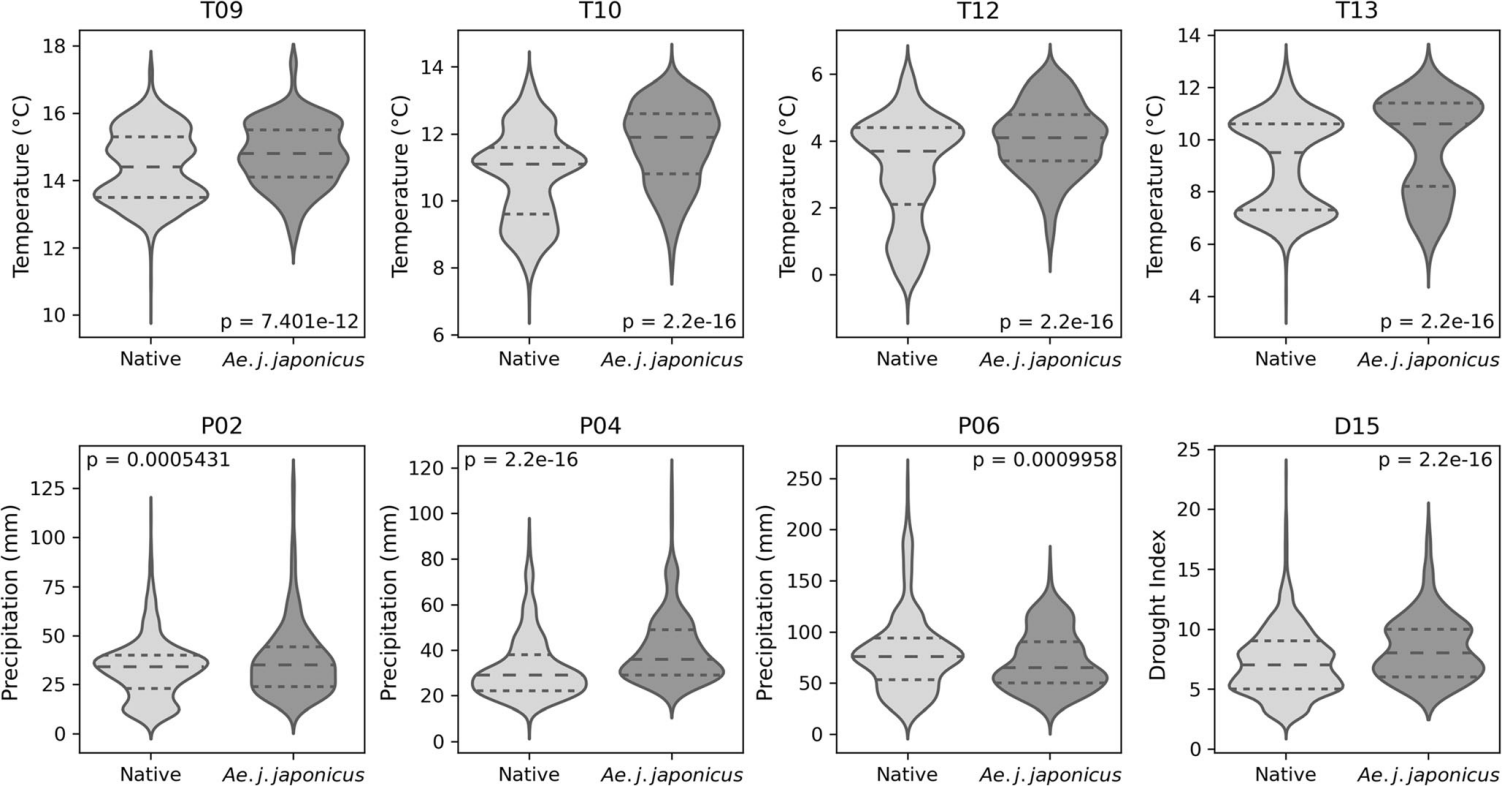
Weather conditions at the mosquito collection sites in the corresponding year of monitoring (between 2011 and 2015) classified by the training group. *Aedes vexans*, *Aedes geniculatus* and *Anopheles daciae* are grouped under the term “Native”. T09 = mean temperature in September, T10 = mean temperature in October, T12 = mean temperature in December, T13 = mean temperature in spring (average of March, April and May), P02 = sum of precipitation in February, P04 = sum of precipitation in April, P06 = sum of precipitation in June, D15 = drought index of autumn (average of September, October and November). This image was created under Python 3.7

All eight weather variables show overlapping areas for both training groups, i.e. the native species and *Ae j. japonicus* (Fig. 3). However, with respect to all weather variables, the distributions differ significantly from each other with *p* < 0.02.

### Model validation

#### Classification

The combination of our training algorithm and the chosen weather variables yielded very good classification results. As can be seen in the confusion matrix (Table 1), 241 of 308 test points (78.2%) on the map, verified to be climatically suitable areas for *Ae. j. japonicus* in 2015, are correctly evaluated (true positives). Conversely, only 67 test points (21.8%) where *Ae. j. japonicus* was present lay in the area categorised to belong to the class of native species. From the target class of native species supported by 115 test points, only 20.9% were predicted to belong to the class of *Ae. j. japonicus*.

**Table 1:** Confusion matrix for the trained model and validation data from 2015

		Predicted class		Sum
		***Ae. j. japonicus***	Native species	
Observed class	***Ae. j. japonicus***	241 (**TP**, TN)	67 (**FN**, FP)	308
	Native species	24 (**FP**, FN)	91 (**TN**, TP)	115
Sum		265	158	423

*TP* true positive, *TN* true negative, *FN* false negative, *FP* false positive (first place and bold referring to *Ae. j. japonicus*, in second place referring to the class of native species)

Regarding precision, recall and *f*1 score, the model classifies the test dataset of *Ae. j. japonicus* extremely well and only slightly less well for the test data of the native species. The *f*1 score in total (weighted average) was 79% (Table 2).

**Table 2:** Validation of the model training with test data from 2015

Class	Precision	Recall	*f*1
*Ae. j. japonicus*	0.91	0.78	0.84
Native species	0.58	0.79	0.67
Total	0.82	0.78	0.79

#### Predictive power

**Fig. 4:**
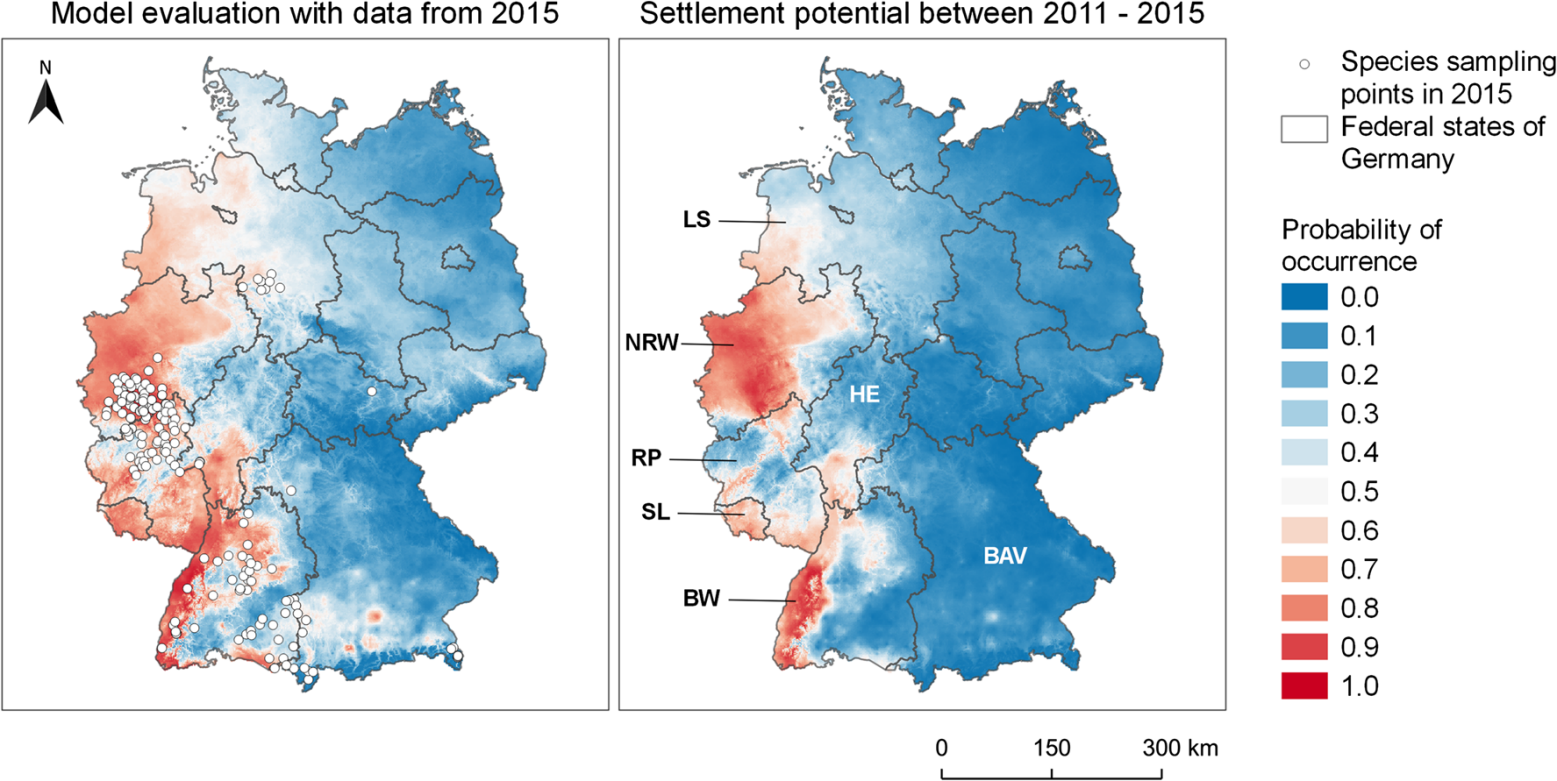
Left: predicted occurrence areas of *Ae. j. japonicus* for the year 2015 as opposed to field samplings in 2015. Species data from 2015 were not included in the model training. Right: Average colonisation potential in the period 01/2011–12/2015 (labelled federal states: LS, Lower Saxony; NRW, North Rhine-Westphalia; HE, Hesse; RP, Rhineland-Palatinate; SL, Saarland; BW, Baden-Wuerttemberg; BAV, Bavaria). This image was created with QGIS 3.8.2

**Fig. 5:**
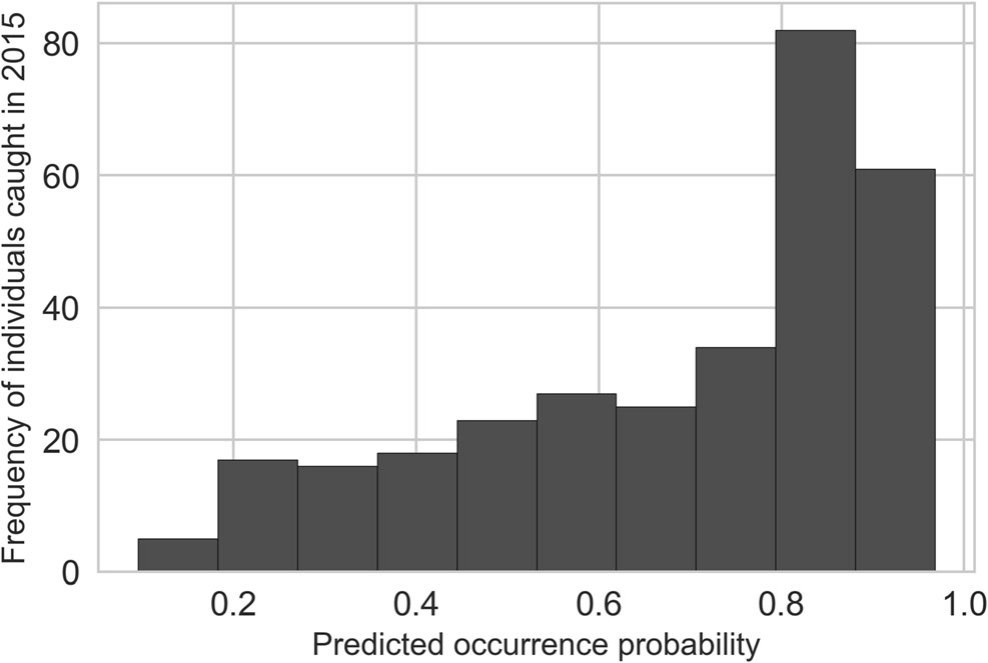
Predicted occurrence probabilities of *Ae. j. japonicus* in Germany, related to weather conditions of 2015, compared with field collections in 2015. This image was created with Python 2.7

A comparison of the predicted occurrence probability for *Ae. j. japonicus* using the weather conditions and the species sampling data from 2015 indicates a high degree of accuracy of the model. This also becomes apparent in the prediction map for the year 2015 (Fig. 4). The occurrence probabilities for the validation points (*n* = 308) have a median of 0.78 and lie in the interquartile range between 0.52 and 0.81 (Fig. 5).

#### Colonisation potential between 2011 and 2015

According to the average climate conditions of the period 2011–2015 (Fig. 4, right), the highest colonisation potentials for *Ae. j. japonicus* (80–100%) lie in the West German federal states of North Rhine-Westphalia (NRW), Baden-Wuerttemberg (BW), Saarland (SL) and in some parts of Rhineland-Palatinate and Hesse. In addition, there is a highly probable distribution area (60–80%) in the southwest of Lower Saxony (LS).

## Discussion

The comparison of the prediction map for the year 2015 with field collection data from 2015 (which have not been included in the model training) showed an extremely good correspondence. The concentration of predicted suitable regions for *Ae. j. japonicus* in western Germany (Baden-Wuerttemberg, Rhineland-Palatinate, Hesse, North Rhine-Westphalia) is not surprising, as we had a high amount of training data from these regions. However, the high occurrence probabilities in Bavaria (south-eastern Germany) under the weather conditions of the year 2015 is remarkable, as is the predicted spread far into the most northern regions of Germany. Indeed, *Ae. j. japonicus* specimens were not only found in 2015 in an area of roughly 900 km^2^ in south-eastern Bavaria on the border to Austria (Zielke et al. 2016), but this species was actually even more widespread in 2016 and 2017 in large areas of southern Bavaria (Kampen et al. 2017; Koban et al. 2019).

The model application to the average climate conditions of the period 2011–2015 shows where good colonisation conditions had been since an early invasion stage during the entire period. We refer to this short period because *Aedes j. japonicus* was discovered in southern Germany (Baden-Wuerttemberg) only in 2008 and in western and northern Germany in 2012. However, in order to make predictions for a mean climate based on a longer period, the model has to be re-calibrated. With the result of long-term averaged climate conditions, it is possible to predict future developments of *Ae. j. japonicus* in Germany. The calibration and forecast for the future period of 2021–2050 are presented by Kerkow et al. (2019).

According to the map for the average climate conditions from 2011 to 2015, the climate in north-eastern Germany was less suitable than in 2015 alone. The suitable area in Lower Saxony is clearly smaller and the prediction values were low (around 10–50%) where the population was located. This is consistent with the observation that the population seems to have decreased over the period 2011–2014, but has expanded again since 2015 (Koban et al. 2019).

In order to better assess the effectiveness of our modelling technique, we compared our results for the averaged climate conditions with those of two other models for Germany produced in completely different ways: (i) Melaun et al. (2015) used occurrence and weather data for 2011–2013 and seven different weather variables to train a MaxEnt model using simulated background data. The result is mapped to the climate of the period 1950–2000 and differs only slightly from ours. A completely different approach (ii) was carried out by Wieser et al. (2019). Using experimental life history data obtained from *Ae. j. japonicus* specimens from southern Germany (temperature-dependent development, mortality, reproduction and density-dependent larval mortality rates), the authors modelled population dynamics for two specific locations and determined where the species can establish stable populations in Germany. The analysis, based on climate data from 1993 to 2013, reveals that the range may be larger than we have modelled. In detail, the habitats in southwestern and western Germany have a similar pattern, but the size of the suitable area in northern Germany is much larger, reaching to the far north and eastwards as far as Berlin.

This leads us to conclude that our model may underestimate the possible spread of *Ae. j. japonicus* in Germany. Although we have already used occurrence data of four years for the training, they may not yet be sufficient for all suitable climatic conditions. However, our result is remarkably congruent with that of the MaxEnt model and thus able to compete with a well-established and widely used method.

A possible problem with data-driven species distribution models, apart from the possibility that not all environmental conditions are reflected, is the quality of the training data. In our case, 70% of our data originate from the citizen science project “Mueckenatlas”. Thus, the modelling results may be slightly biased, as the majority of mosquitoes submitted by the participants are most likely from densely populated regions. However, about 30% of the training data originate from systematic field collections which particularly concentrated on the margins and surroundings of known distribution areas of *Ae. j. japonicus* (Kampen et al. 2016a).

Regarding the selection of mosquito species to replace absence data in machine learning, the question arises as to why we chose the species *Ae. vexans*, *Ae. geniculatus* and *An. daciae*. Our modelling approach is driven solely by climate data, and the aim was to determine the climatic-ecological niche of the Asian bush mosquito. We therefore searched for mosquito species, which, when considering some relevant climate parameters (mean annual and summer precipitation, drought indices and temperature data), seemed to differ in their occurrence spectrum from that of *Ae. j. japonicus*. We have tested different species combinations in the initial phase of modelling and found, firstly, that the use of more or less than three species tends to diminish the results measured by the total *f*1 score. Secondly, the combination of the two common species *Ae. vexans* and *Ae. geniculatus* together with the species *An. daciae*, which presumably prefers more continental conditions, achieved particularly good results. In a later modelling phase, we optimised the selection of climate variables by means of a deep learning method (Wieland et al. 2017). The existence of competitive conditions was not a criterion for the selection of the species. Competitive situations for mosquitoes result from oviposition and development of pre-imaginal stages in the same habitats. Amongst our species selected for modelling, only the dendrolimnobiotic species *Ae. geniculatus* (Becker et al. 2011) occasionally lays its eggs in the same water sources as *Ae. j. japonicus* (Damiens et al. 2014; Seidel et al. 2016).

Another interesting aspect of our result is that our model performs very well in validation, although the weather and climate data of the two training classes “native species” and “*Ae. j. japonicus*” are noisy and partly overlap (Fig. 4). Consequently, the support vector machine was able to handle this circumstance effectively. This is probably due to the use of the RBF kernel, which can identify complicated distribution patterns in the data. When comparing different algorithms and their combinations for our study (Früh et al. 2018), this algorithm also achieved the best result of all individually applied algorithms. However, if more mosquito data become available in the future via the citizen science project, we would consider using learning algorithms that are much more powerful in general, such as XG-Boost (Chen and Guestrin 2016; Brownlee 2017). The main strength of the support vector machine lies in the handling of smaller amounts of data (Pedregosa et al. 2011; Nalepa and Kawulok 2019).

As mentioned before, mesoclimate data with a resolution of 1 km^2^ can only give an approximate picture of the suitable habitats and thus a possible distribution of a mosquito species. At the local level, landscape forms and the resulting microhabitats also play a role. It is also possible that unfavourable climatic conditions may be compensated in some way. For example, in regions with low precipitation, people may regularly provide water-filled breeding habitats such as rain water barrels or flowerpots. Therefore, in a further step, the results of this model were combined with landscape and wind data, which led to a significant improvement of the results as measured by the hit rate for *Ae. j. japonicus* (Kerkow et al. 2019).

## Conclusions

Our model approach appears to be suitable for predicting the distribution area of the Asian bush mosquito *Ae. j. japonicus* in newly invaded areas. The results were achieved by the exclusive use of presence data of this species and three native species. The model output matches extremely well with presence data from 2015, which was not included in the model training. Due to the use of presence-only data, the method presented here is well-suited to datasets that are based on passive monitoring programmes such as citizen science projects. Our result is consistent with that of another data-driven machine-learning approach that used generated absence data (Melaun et al. 2015). However, when applying the method, it is important to bear in mind that the dispersal potential of an invasive species is underestimated if the dataset is too small to represent the realised ecological niche of a species.

With the help of large-scale, long-term averaged climate data, only a rough impression of the possible further spread of invasive mosquito species in Germany can be obtained, independent of the modelling technique. On a smaller scale, land use forms correlating with the presence of certain breeding habitats and causing certain microclimates have an additional important impact.

## Data Availability

Mosquito occurrence data used in this model are provided in the supplementary information files. We uploaded four Excel tables, one for each mosquito species, containing the year of discovery, the geo-coordinates and the weather values relevant for both model training and validation. The CULBASE database is not yet publicly accessible, further mosquito data are available on reasonable request to DW or HK.
